# Genetic variants influenced the risk of bleeding and pharmacodynamics of rivaroxaban in patients with nonvalvular atrial fibrillation: A multicentre prospective cohort study

**DOI:** 10.1002/ctm2.1263

**Published:** 2023-05-18

**Authors:** Qian Xiang, Zhe Wang, Guangyan Mu, Qiufen Xie, Zhiyan Liu, Shuang Zhou, Hanxu Zhang, Zining Wang, Jie Jiang, Kun Hu, Yatong Zhang, Zinan Zhao, Dongdong Yuan, Liping Guo, Tingting Wu, Jinhua Zhang, Na Wang, Jing Xiang, Zhichun Gu, Jianjun Sun, Yimin Cui

**Affiliations:** ^1^ Department of Pharmacy Peking University First Hospital Beijing China; ^2^ School of Pharmaceutical Sciences Peking University Health Science Center Beijing China; ^3^ Department of Cardiology Peking University First Hospital Beijing China; ^4^ Department of Pharmacy Beijing Hospital Beijing China; ^5^ Department of Pharmacy Zhengzhou Seventh People's Hospital Zhengzhou China; ^6^ Department of Pharmacy Fujian Medical University Union Hospital Fuzhou China; ^7^ Department of Pharmacy The Second affiliated Hospital of Chongqing Medical University Chongqing China; ^8^ Department of Pharmacy Ren Ji Hospital Shanghai Jiao Tong University School of Medicine Shanghai China; ^9^ Department of Pharmacy affiliated Hospital of Inner Mongolia Medical University China; ^10^ Institute of Clinical Pharmacology Peking University Beijing China

**Keywords:** atrial fibrillation, bleeding, genetic polymorphism, pharmacodynamics, rivaroxaban

## Abstract

**Introduction:**

Individual variability of rivaroxaban was observed in clinical application. This study aimed to identify genetic variants associated with the variability of pharmacodynamics and bleeding risk of rivaroxbaban in patients with nonvalvular atrial fibrillation (NVAF).

**Materials and Methods:**

From June 2017, and July 2019, this study enrolled 257 patients with NVAF receiving rivaroxaban. Pharmacodynamics was assessed by determining anti‐Factor Xa (anti‐FXa) level 3 h after rivaroxaban administration as peak concentration. Whole‐exome sequencing was performed to detected single nucleotide polymorphisms (SNPs). This study was registered (NCT03161496).

**Results:**

The bleeding events within 12 months were significantly related to the peak anti‐FXa level (*p* = .027). *SUSD3* rs76292544 was associated with 12‐month bleeding events (odds ratio [OR]: 4.20, 95% confidence interval [CI]: 2.17–8.14, *p* = 6.43×10^−5^). Five SNPs including *NCMAP* rs4553122 (*p* = 2.29×10^−5^), *PRF1* rs885821 (*p* = 7.02×10^−5^), *PRKAG2* rs12703159 (*p* = 7.97×10^−5^), *PRKAG2* rs13224758 (*p* = 8.70×10^−5^), and *POU2F3* rs2298579 (*p* = 8.24×10^−5^) were associated with peak anti‐FXa level. Genetic variants of 52 SNPs from 36 genes including *GOT2* rs14221 and *MMP13* rs640198 were potentially related to 12‐month bleeding events caused by rivaroxaban's efficacy.

**Conclusions:**

Peak anti‐FXa level was associated with risk of bleeding events in patients with NVAF receiving rivaroxaban. *SUSD3* rs76292544 was suggestively associated with 12‐month bleeding events and five SNPs (*NCMAP* rs4553122, *PRF1* rs885821, *PRKAG2* rs12703159, rs13224758 and *POU2F3* rs2298579) were suggestively associated with peak anti‐FXa level.

## Graphical abstract

Peak anti‐Factor Xa level was associated with bleeding events of rivaroxaban.

Minor allele carriers of *SUSD3* rs76292544 increased bleeding risk.

Genetic variants of five SNPs, including *NCMAP* rs4553122, *PRF1* rs885821, *PRKAG2* rs12703159, *PRKAG2* rs13224758 and *POU2F3* rs2298579 were associated with peak anti‐FXa level.

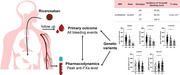

## 1 INTRODUCTION

Direct oral anticoagulants, such as rivaroxaban, are mostly used to treat patients at high risk of thromboses, such as those with nonvalvular atrial fibrillation (NVAF) and venous thromboembolism. Rivaroxaban inhibits factor Xa, blocking the common pathway of coagulation cascades. Although rivaroxaban reduces the incidence of ischaemic events, it has been linked to an increased risk of major bleeding events from a gastrointestinal site in clinical applications.^1^ Previous studies have reported that in patients using rivaroxaban, the incidence of any bleeding events ranged from 14.9% to 18.0% per year, with the incidence of major bleeding events ranging from 3.0% to 3.6%,^1^, ^2^ posing risks of hospitalisation and death.

Individual variations in the pharmacodynamics and bleeding complications of rivaroxaban have been reported in clinical application.^3^, ^4^, ^5^, ^6^, ^7^, ^8^, ^9^ For instance, East Asian patients with AF exhibited a higher mean peak drug level (228.0–386.2 ng/mL)^3^, ^4^ than Caucasian patients (161.7–294.4 ng/mL).^5^ In addition, the peak plasma levels of rivaroxaban were associated with the risk of bleeding.^6^, ^7^ Furthermore, East Asian patients demonstrated higher risk of bleeding events than Caucasian patients.^8^, ^9^ The incidence of major or nonmajor clinically relevant bleeding events was 20.9% and 14.5% in East Asian and non‐East Asian patients, respectively.^8^

The relation between genetic polymorphisms and rivaroxaban pharmacodynamics or clinical outcomes has been studied before.^10^, ^11^, ^12^, ^13^ Previous research on rivaroxaban has detected the relationship of genetic variations in drug‐metabolising enzymes and transporters with rivaroxaban pharmacodynamics in patients with AF. Single nucleotide polymorphisms (SNPs) of the *ATP Binding Cassette Subfamily B Member 1 (ABCB1)* were linked to the differences in rivaroxaban area under the curve^11^ and trough concentrations.^12^ However, these SNPs were not statistically associated with bleeding events.^12^, ^13^ Research into genes other than drug‐metabolising enzymes and transporters in terms of pharmacodynamics and bleeding has received little attention. Therefore, in this multicente prospective cohort study, whole‐exome sequencing was conducted to explore potential genes associated with pharmacodynamics and bleeding events after rivaroxaban therapy in patients with NVAF.

## 2 PATIENTS AND METHODS

### 2.1 Study design

This was a prospective cohort study in multicentre. The study protocol was approved by the independent ethics committee of Peking University First Hospital and all subcentral hospitals with the trial registration number of NCT03161496. This study follows the principles of the Declaration of Helsinki. All patients who participated in this study provided informed consent.

Potential participants were included with inclusion criteria as follows: (a) patients of age over 18; (b) patients diagnosed as NVAF; (c) patients received rivaroxaban as anticoagulation therapy; (d) patients provided written informed consent. The exclusion criteria were as follows: (a) patients with immunodeficiency diseases, viral hepatitis, severely abnormal liver function, or renal function; (b) patients receiving a combined therapy of P‐glycoprotein inhibitors or inducers, such as itraconazole, ketoconazole, voriconazole, posaconazole, ritonavir, rifampicin, phenytoin, phenobarbital, and carbamazepine, within 14 days before rivaroxaban application; (c) any rivaroxaban contraindications, such as active bleeding, previous history of intracranial, hypersensitivity or gastrointestinal haemorrhage within 6 months, and any major operations within 30 days.

### 2.2 Genetic testing

During the follow‐up period, blood samples were collected for DNA preparation and genotyping. SureSelect target enrichment system and Illumina NovaSeq 6000 sequencer (Illumina, United States) were used to detected SNPs. With 200bp extensions on either end, a total of 341 919 SNPs were detected in exons of variant filtering and prediction. SNPs were included with criteria as follows: missing rate less than 10%, minor allele frequency more than 5% and Hardy‐Weinberg equilibrium *p*‐value more than 10^–6^. Finally, 75 853 SNPs were included for correlation analysis.

### 2.3 Study endpoints

Follow‐up visits were made by phone or during routine clinic visits 1 month, 6 months, 1 year and 2 years after enrolment. The primary safety outcome was bleeding events, including major and minor bleeding as classified by the Bleeding Academic Research Consortium (BARC).^14^ The secondary endpoint was stroke or systemic embolism events. Based on the abrupt onset of localised pain and imaging examinations, systemic embolism events were diagnosed at sites other than in the central nervous system causing acute ischemia.^15^

### 2.4 Pharmacodynamics’ testing

After the blood drug concentration reached a steady‐state (taking rivaroxaban for more than 48 h), blood samples were collected at 3 h after rivaroxaban administration as peak concentration.

The blood samples were centrifuged and peak anti‐factor Xa (anti‐FXa) level was measured on the Sysmex® CS‐2100i (Sysmex, Kobe, Japan) using anti‐FXa kit (BIOPHEN DiXaI®, HYPHEN BioMed, France) with measuring range 0–500 ng/mL, variance in intra‐test 1.5% and variance in inter‐test 2.3%. Rivaroxaban calibrators and controls were used to ensure the quality of tests.

### 2.5 Statistical analyses

Continuous variables were presented as mean and standard deviation, whereas categorical variables were presented as frequencies with percentages. Association analyses of genetic variations with peak anti‐FXa level and bleeding events within 12 months were performed in PLINK v1.09 and R software^16^ by linear regression and logistic regression with Bonferroni adjustment respectively, assuming an additive genetic model. SNPs association analyses with peak anti‐FXa level were adjusted for sex, age, dose of rivaroxaban and MPV. SNPs association analyses with bleeding events were adjusted for MPV and HAS‐BLED score. Predictors associated with bleeding events or peak anti‐FXa level at *p* < .2 in univariable analysis and remained statistically significant (*p* < .05) at that level after multivariable adjustment were chosen^17^ (Supplementary Appendix). Baseline characteristics previously reported to affect the peak anti‐FXa level and clinical outcomes of rivaroxaban were included in the analyses.^18^, ^19^ Continuous variables were analysed the log‐normally distribution by Kolmogorov‐Smirnov test. Chi‐square test and nonparametric test was used for correlation analysis of variables with or without normal distribution respectively. By Bonferroni adjustment, statistically significant threshold of *p*‐value was .05/75 853 = 6.59×10^−7^. In addition, according to prior published researches (Supplementary Appendix), three candidate genes including *ABCB1*, *ABCA6* and *APOB* were analysed the association with peak anti‐FXa level and bleeding events of rivaroxaban.

SNPs associated with both peak anti‐FXa level and bleeding events within 12 months were selected to explore genes potentially related to bleeding events caused by rivaroxaban's high efficacy (*p* < .05). In addition, the beta value of PLINK analysis was screened to select SNPs with a consistent influence on peak anti‐FXa level and bleeding events. Using logistical regression, a gene‐based prediction model was determined. And its predictive effect for bleeding events was evaluated by receiver operating characteristic (ROC) curves. In addition, using Genotype‐Tissue Expression (GTEx) (https://gtexportal.org),^20^ quantitative trait locus analyses was conducted to identify potential effect of SNPs mutation.

## 3 RESULTS

### 3.1 Baseline characteristics

A total of 257 patients with NVAF were enrolled between June 2017, and July 2019 (Supplementary Appendix). At the time of enrolment, 50.97% (131/257) of patients had radiofrequency catheter ablation (RFCA). The baseline characteristics of the included patients are shown in **Table** **1**.

**TABLE 1:** Baseline characteristics of participants.

Characteristics	Rivaroxaban (*N* = 257)
Age, year	68.9 ± 10.4
Male sex, no. (%)	133 (51.8%)
Body mass index	25.3 ± 3.8
Weight, kg	69.7 ± 14.1
Rivaroxaban dose, no.(%)	
5 mg	1 (0.4%)
10 mg	30 (11.7%)
15 mg	91 (35.4%)
20 mg	135 (52.5%)
Smoking state, no.(%)	
No smoking	193 (75.1%)
Current smoking	33 (12.8%)
Former smoking	31 (12.1%)
Radiofrequency catheter ablation, no. (%)	131 (51.0%)
Laboratory tests	
Mean platelet volume, fL	10.2 ± 1.5
Creatinine clearance, mL/min	81.0 ± 21.7
Comorbidities, no.(%)	
Hypertension	176 (68.5%)
Diabetes mellitus	66 (25.7%)
Hyperlipidemia	76 (29.6%)
Coronary heart disease	86 (33.5%)
Heart faliure	36 (14.0%)
Stroke	46 (17.9%)
Concomitant medication, no.(%)	
Antiplatelet drugs	52 (20.3%)
Angiotensin receptor blocker/angiotensin converting enzyme inhibitor	114 (44.4%)
Calcium channel blocker	87 (33.9%)
Statin	119 (46.3%)
β blocker	137 (53.3%)
Proton pump inhibitors	53 (20.6 %)
HAS‐BLED score	1.8 ± 1.1
CHA_2_DS_2_‐VASc score	3.1 ± 1.6

### 3.2 Peak anti‐FXa test and clinical outcomes

Peak anti‐FXa testing was conducted on 251 patients with NVAF and 6 patients refused the peak anti‐FXa testing due to the limitation of the time for drug administration and arrival at clinic. The mean anti‐FXa level was 273.38 ± 130.86 ng/mL.

Final follow‐up visits occurred between October 2017, and July 2021. The average follow‐up time was 13.16 months. In follow‐up, 0.78%, 1.18%, 3.85% and 3.31% of patients lost at 1, 6, 12 and 24 months, respectively. Adjustment of clinical treatment strategies was the most common reason for discontinuation, accounting for 94.1% (16/17), 88.5% (54/61), 57.7% (15/26) and 67.9% (47/52) at 1, 6, 12 and 24 months, respectively.

A total of 11.4% (29/1254), 13.5% (28/208), 15.2% (23/151) and 14.6% (13/89) patients suffered from bleeding events at 0–1, 2–6, 7–12 and 13–24 months. Within 24 months, bleeding events mostly occurred in gingival, accounting for 40.9% of all bleeding events. During follow‐up, 12 patients experienced composite efficacy events, including nine strokes and three systemic embolism events. A detailed description of clinical outcomes during follow‐up is shown in **Table** **2**.

**TABLE 2:** Clinical outcomes within 1, 6, 12 and 24 months follow‐up.

	0‐1 month (*N* = 254) no. (%)	2‐6 months (*N* = 208) no. (%)	7‐12 months (*N* = 151) no. (%)	13‐24 months (*N* = 94) no. (%)
**Bleeding events**	29 (11.4%)	28 (13.5%)	23 (15.2%)	13 (13.8%)
Type 1	26 (10.2%)	26 (12.5%)	18 (11.9%)	12 (12.8%)
Type 2	3 (1.2%)	1 (0.5%)	5 (3.3%)	1 (1.1%)
Type 3	0 (0.0%)	1 (0.5%)	0 (0.0%)	0 (0.0%)
Subcutaneous bleeding	9 (3.5%)	8 (3.8%)	7 (4.6%)	5 (5.3%)
Intraocular bleeding	1 (0.4%)	2 (0.5%)	1 (1.3%)	0 (0.0%)
Gingival bleeding	12 (4.7%)	15 (7.2%)	7 (4.6%)	4 (4.3%)
Epistaxis bleeding	1 (0.4%)	3 (1.4%)	2 (1.3%)	2 (2.1%)
Gastrointestinal bleeding	4 (1.6%)	7 (3.4%)	4 (2.6%)	1 (1.1%)
Urinary bleeding	2 (0.8%)	1 (0.5%)	1 (0.7%)	0 (0.0%)
Vaginal bleeding	1 (0.4%)	0 (0.0%)	1 (0.7%)	0 (0.0%)
**Stroke or systemic embolism events**	0 (0.0%)	5 (2.4%)	6 (4.0%)	2 (2.1%)
Stroke	0 (0.0%)	5 (2.4%)	4 (2.6%)	2 (2.1%)
Systemic embolism events	0 (0.0%)	0 (0.0%)	2 (1.3%)	1 (1.1%)

Within 12 months, 34.3% patients with NVAF suffered from bleeding events. The incidence of bleeding events were significantly related to the peak anti‐FXa level, which were significantly increased in patients with bleeding events than in those without (309.53 ng/mL vs. 276.11 ng/mL, *p* = .027, respectively).

### 3.3 Genetic association analysis

**FIGURE 1:**
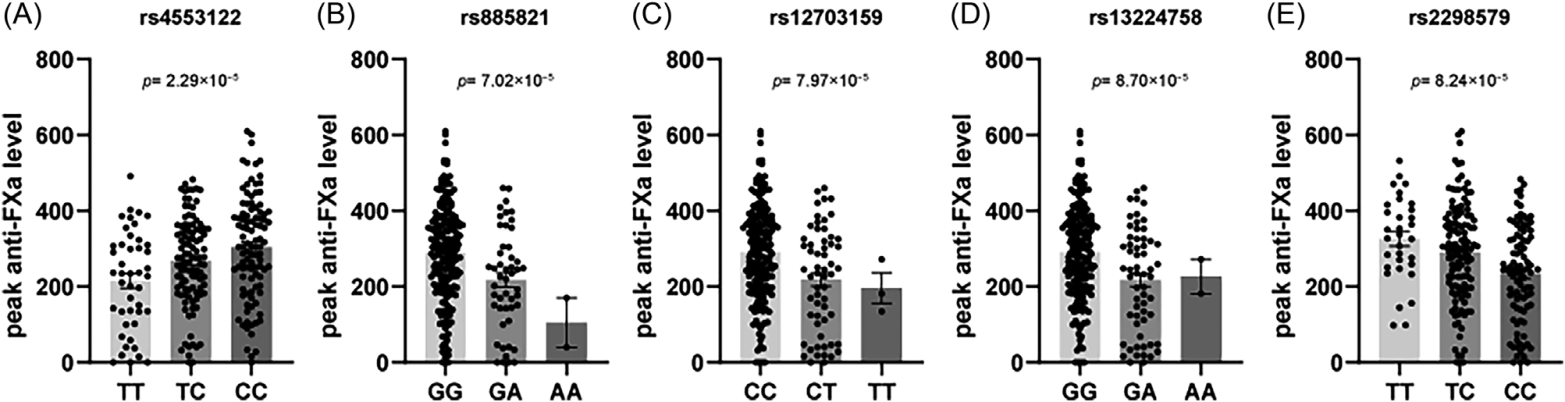
Suggestive genetic variations of peak anti‐FXa level. Annotation: A. *NCMAP* rs4553122; B. *PRF1* rs885821; C. *PRKAG2* rs12703159; D. *PRKAG2* rs13224758; E. *POU2F3* rs2298579.

Given the incidence rates and sample size of this study, for screening suggestive genetic variations of 12‐month bleeding events and peak anti‐FXa level, the *p*‐value significant threshold was relaxed to 1.0×10^−4^. For bleeding events, *SUSD3* rs76292544 was related with bleeding events through 12 months (odds ratio [OR]: 4.20, 95% confidence interval [CI]:2.17–8.14, *p* = 6.43×10^−5^). For peak anti‐FXa level, there were five suggestive SNPs, including *NCMAP* rs4553122 (*p* = 2.29×10^−5^), *PRF1* rs885821 (*p* = 7.02×10^−5^), *PRKAG2* rs12703159 (*p* = 7.97×10^−5^), rs13224758 (*p* = 8.70×10^−5^) and *POU2F3* rs2298579 (*p* = 8.24×10^−5^). The detailed description of SNPs is provided in **Table** **3**. The *NCMAP* rs4553122 variant was the most strongly associated with peak anti‐FXa level. The anti‐FXa levels were statistically higher in CC mutant carriers than TT carriers (305.8 ng/mL vs. 212.7 ng/mL) as shown in **Figure** **1**.

**TABLE 3:** Effects of suggestive genetic variations on peak anti‐FXa level and 12‐month bleeding events of rivaroxaban.

SNP	Allele	Gene	Participant (WH/HTZ/MT)	Peak anti‐Xa/ng/mL (WH/HTZ/MT)	*p* Value (peak anti‐FXa)	Incidence of 12‐month bleeding event (WH/HTZ/MT)	*p* value (12‐month bleeding events)
rs76292544	A>T	*SUSD3*	188/57/5	279.5/250.8/307.0	.552	25.8%/55.0%/100%	6.43×10^−5^
rs4553122	T>C	*NCMAP*	44/105/101	212.7/268.0/305.8	2.29×10^−5^	38.7%/32.0%/35.2%	.859
rs885821	G>A	*PRF1*	200/48/2	288.3/220.5/70.3	7.02×10^−5^	35.3%/31.6%/*NA*	.920
rs12703159	C>T	*PRKAG2*	188/59/3	292.0/218.6/196.4	7.97×10^−5^	35.6%/32.6%/0%	.524
rs13224758	G>A	*PRKAG2*	188/60/2	292.0/217.2/227.1	8.70×10^−5^	35.6%/32.6%/0%	.524
rs2298579	T>C	*POU2F3*	32/123/96	326.8/291.1/232.9	8.24×10^−5^	20.8%/45.3%/22.4%	.480

Abbreviations: MH, mutated homozygous; HTZ, heterozygous; WH, wild homozygous.

Candidate genes *ABCB1* rs1045642, *ABCB1* rs2032582, *ABCA6* rs13306198 and *APOB* rs7212506 were detected in present study. The association analyses of genetic variant between peak anti‐FXa levels and incidence of 12‐month bleeding events were shown in Supplementary Appendix. Only *ABCA6* rs13306198 variant was associated with incidence of 12‐month bleeding events (*p* = .024).

### 3.4 Suggestive genetic variations associated with the risk of bleeding by rivaroxaban pharmacodynamics

To screen potential genetic variations associated with bleeding events directly caused by rivaroxaban administration, the *p*‐value selection threshold was relaxed to .05 for screening SNPs related to both peak anti‐FXa level and bleeding events within 12 months were screened to identify genes. In total, 179 SNPs in 120 genes were detected to be related with peak anti‐FXa level and 12‐month bleeding events. In addition, 52 SNPs from 36 genes were selected for their consistent influence on the peak anti‐FXa level and bleeding events as shown in Supplementary Material.

Among these SNPs, the strongest association was found between *MMP13* rs640198 and the incidence of bleeding events (*p* = .0011). GG carriers of rs6728818 had a lower 12‐month bleeding rate than TT carriers (13.9% vs. 49.1%). Patients with GG genotypes had a lower 12‐month bleeding rate compared to AA and AG genotypes (OR: 2.27, 95%CI: 1.41–3.67). The *GOT2* rs14221 variant was the most strongly associated with peak anti‐FXa level (*p* = .0053). The anti‐FXa levels was statistically lower in CC mutant carriers than AA carriers (257.8 ng/mL vs. 314.0 ng/mL) with a lower bleeding rate of CC carriers during 12‐month follow‐up period (25.0% vs. 41.0%).

### 3.5 Expression quantitative trait loci analyses

The putative functional relevance of genes in whole blood was evaluated by GTEx database, as shown in Supplementary Material. In whole blood, samples with rs76292544 A genotype had significantly higher level of alternative splicing of SUSD3 mRNA than samples with rs76292544 T genotype (*p* = 5.2×10^−12^, normalised effect size [NES] = –0.72). C genotype of rs14221 had significantly lower GOT2 mRNA levels in the whole blood than samples with rs14221 A genotype (*p* = 2.4×10^−15^, NES = –0.12). C genotype of rs30842, A genotype of rs8106303, G genotype of rs7252937, T genotype of rs8110220, C genotype of rs3736456, C genotype of rs28540767, C genotype of rs1130529 and G genotype of rs38414 was associated with increased expression of corresponding mRNA in whole blood.

### 3.6 Gene‐based prediction model

**FIGURE 2:**
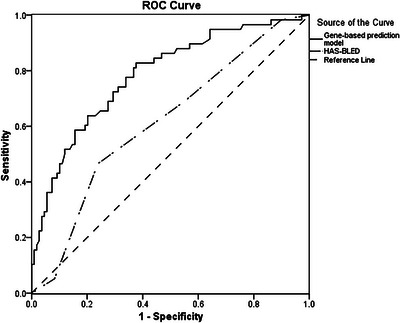
Receiver operating characteristic curve of gene‐based prediction model included MPV, rs76292544, rs2255317, and rs6728818. Annotation: The area under the curve (AUC) of gene‐based prediction model ROC was 0.784 with sensitivity of 82.8% and specificity of 62.4%. The AUC of HAS‐BLED was 0.614.

After logistical regression, MPV, rs76292544, rs2255317 and rs6728818 were included to form a gene‐based prediction model. The sensitivity and specificity of prediction for 12‐month bleeding events were 82.8% and 62.4%, respectively. The area under the curve (AUC) of ROC was 0.784, as provided in **Figure** **2**.

## 4 DISCUSSION

This study analysed the genetic polymorphisms for peak anti‐FXa level and bleeding events in patients with NVAF. The peak anti‐FXa level was found to be significantly associated with 12‐month bleeding events. We first identified suggestive *SUSD3* rs76292544 associated with 12‐month bleeding events and five SNPs (*NCMAP* rs4553122, *PRF1* rs885821, *PRKAG2* rs12703159, rs13224758 and *POU2F3* rs2298579) associated with peak anti‐FXa level. Genetic variants including *GOT2* rs14221 and *MMP13* rs640198 were potentially related to 12‐month bleeding events caused by rivaroxaban's high efficacy.

The rivaroxaban concentration could be detected via anti‐FXa level and was related with clinical bleeding events. The plasma rivaroxaban concentration presented a linear correlation with anti‐FXa level (Pearson's correlation coefficient: 0.98).^21^ Additionally, peak anti‐FXa level was reported to associate with bleeding events. Previous studies reported that patients with bleeding complications showed higher peak anti‐FXa level in the follow‐up period than those without bleeding events (2.40 vs. 1.84 IU/mL, *p* = .001^7^; 1.93 vs. 1.35 IU/mL, *p* < 0.01^22^). Additionally, compared with the peak anti‐FXa control, the anti‐FXa level of patients at the time of a bleeding event tended to be higher (245.9 vs. 177.6 ng/mL, *p* = .13).^23^ Herein, bleeding events were significantly related to peak anti‐FXa level (*p* = .027), which further confirmed the relationship of peak anti‐FXa level and bleeding risk in patients using rivaroxaban.

In the present study, 34.3% patients with NVAF suffered from bleeding events within 12 months. Previous study reported that the incidence of major or nonmajor clinically relevant bleeding events was 14.5% in non‐East Asian patients,^8^ which was lower than East Asian patients. The active metabolites were also 20–30% lower in Caucasians vs. Japanese.^24^ This is known as “East Paradox”: compared with Caucasian patients, East Asians have lower ischaemic risk and higher bleeding risk in anticoagulant therapy.^25^ The higher propensity of bleeding in East Asians may be caused by multiple factors including lower body weight, genetic variants and higher prevalence of bleeding relevant comorbidities (such as *Helicobacter pylori* infection^26^ and intracranial atherosclerosis^27^). In present study, among patients suffered from bleeding, gingival, subcutaneous and gastrointestinal bleeding was the most common ones, accounting for 40.9%, 31.2% and 17.2% of all bleeding events respectively. Patients with concomitant gingivitis, gastric ulcers or comedication of nonsteroidal anti‐inflammatory drug increases the risk of gingival or upper gastrointestinal bleeding in patients taking anticoagulants.^28^, ^29^ We did not observe an association of gastric ulcers or aspirin use with gastrointestinal or other types of bleeding events (*p* > .05). The association of Helicobacter pylori infection, gingivitis with bleeding events was not evaluated due to the limitation of medical history collection and needs to assess in future study.

*NCMAP* rs4553122 encodes Noncompact myelin‐associated protein (NCAMP), which participate in myelin formation. Previous study reported that NACMP variants was potentially related with warfarin pharmacodynamics. Warfarin is administered as a racemic mixture of the R‐warfarin and S‐warfarin, which is the more potent enantiomer. In warfarin treated adults, *NACMP* rs55816255 variants decreased the metabolic ratio of S‐warfarin to R‐warfarin (*p* = 2×10^−7^).^30^ Therefore, *NACMP* gene was suggested to associate with pharmacodynamics of warfarin. In present study, compared with TT and TC carriers of *NCMAP* rs4553122, CC carriers increased the peak anti‐FXa level of rivaroxaban by 20.8%. It indicated that *NCMAP* might involve in the variety of rivaroxaban pharmacodynamics.

*SUSD3* rs76292544 encodes Sushi domain‐containing protein 3 (SUSD3). It is a single‐pass membrane protein on plasma membrane. Previous study indicated that SUSD3 may play a role in cardiovascular diseases. *SUSD3* upregulated in coronary heart disease (1.03‐fold change in microarray expression values, *p* = .0080).^31^ And *SUSD3* gene also differential expressed in patients with ischaemic stroke and matched controls.^32^ In this study, *SUSD3* rs76292544 mutant TT genotype significantly increased the bleeding risk of rivaroxaban in 12‐month follow‐up compared with AA and AT carriers (32.9% to 100%). However, variation of *SUSD3* rs76292544 was not related with peak anti‐FXa level (*p* = .552). This suggests that *SUSD3* may affect the bleeding propensity of patients rather than rivaroxaban pharmacodynamics.

Screening potential genetic variations associated with bleeding events directly caused by rivaroxaban administration, *GOT2, MMP13*, and *COL6A3* exhibited suggestive effect on the risk of bleeding by rivaroxaban pharmacodynamics. *GOT2* gene encodes fatty acid binding protein. It is a pyridoxal phosphate‐dependent enzyme, which potentially participated in the uptake or transport of anticoagulant drugs including warfarin.^33^ In this study, *GOT2* rs14221 variant was the most strongly associated with peak anti‐FXa level (*p* = .0053). Compared with AA carriers, rs14221 CC mutant carriers showed lower peak anti‐FXa level by 17.9% and bleeding rate by 40.0%. In GTEx database, the mRNA levels of whole blood samples with rs14221 C genotype was lower than A genotype (*p* = 2.4×10^−15^). The rs14221 mutation most likely reduced the expression of GOT2 and reduced the bleeding risk in rivaroxaban with lower FX activation.

The *MMP13* gene encodes matrix metalloproteinase (MMP)‐13, which participate degradation of extracellular matrix proteins including collagens, fibrinogen, factor XII and fibrillin‐rich microfibrils.^34^ It is associated with anticoagulants and haemorrhage. In primary human cell cultures, administration of rivaroxaban increased MMP‐13 with a 36.3‐fold change (*p* < .05).^35^ In atherosclerotic lesions, activation of MMP‐13 was associated to aggravate collagen breakdown and intra‐plaque haemorrhages.^36^ In the present study, compared with TT wild homozygous, GG genotype of *MMP13* rs640198 increased peak anti‐FXa level by 16.8% and the incidence of bleeding events within 12 months by 1.53‐fold change. Patients with GG genotypes also had a higher incidence of 12‐month bleeding events compared to GT and TT genotypes (OR: 2.27, 95%CI: 1.41–3.67).

The *COL6A3* rs6728818 variant, which is the most significantly related to bleeding events, encodes the collagen alpha‐3 chain. It participates in collagen biosynthesis, which regulates the blood coagulation cascade. Collagen could bind to blood‐circulating FXII and promote FXII activation through conformational change.^37^ FXIIa further activates FX and induces blood coagulation. In this study, GG carriers of *COL6A3* rs6728818 reduced the incidence of 12‐month bleeding events by 28.8% compared with AA carriers. The rs6728818 mutation most likely activates FX and reduces the bleeding risk in rivaroxaban clinical applications.

Previous studies have linked several susceptible genes to rivaroxaban pharmacodynamics or bleeding events, including *ABCB1*, *APOB* and *ABCA6*. The *ABCB1* rs1045642 mutant genotypes had a reduced concentration of direct oral anticoagulants.^11^ However, *ABCB1* genotypes were reported to affect renal secretion of the drug rather than the metabolism of rivaroxaban.^5^ Meanwhile, there was no obvious association between *ABCB1* genotypes and bleeding events.^38^ In this study, the *ABCB1* rs1045642 variant had no statistically significant association with anti‐FXa level or bleeding events over the follow‐up period. The difference may be attributed to the pooled analysis of all direct oral anticoagulants, including dabigatran, edoxaban, apixaban and rivaroxaban, in a previous study. Furthermore, *ABCA6* has been linked to bleeding events of rivaroxaban. In a case‐control study of rivaroxaban‐treated patients, the *ABCA6* rs7212506 variant increased the bleeding risk of rivaroxaban.^39^ In the present study, compared with patients of rs7212506 CT genotype, patients of TT genotype exhibited a reduced incidence rate of bleeding events from 40.6% to 32.4% during 12 months without statistical significance.

In order to explore genetic variants for prediction of bleeding risk in patients treated with rivaroxaban, a gene‐based prediction model including MPV, rs76292544, rs2255317 and rs6728818 was constructed in this study. Compared with HAS‐BLED score, the gene‐based model was more accurate within 1, 6 and 12 months with a higher AUC of ROC. The AUC of gene‐based model and HAS‐BLED score within 1, 6 and 12 months was 0.668 vs. 0.809, 0.623 vs. 0.825 and 0.614 vs. 0.784, respectively. However, considering the availability and expense of genetic test in clinic, the gene‐based model still needs to be further validated and optimised in large cohort to optimise the prediction accuracy and improve cost‐effectiveness.

In addition, HAS‐BLED score had a higher prediction accuracy of bleeding events at 1 month than at 6 and 12 months. It might be caused by the variation of bleeding risk during follow‐up period. Bleeding risk can be classified into modifiable and nonmodifiable factors. Nonmodifiable ones included gender, age, genetic factors, etc. Modifiable ones included comedication (antiplatelet agents and nonsteroidal anti‐inflammatory drugs), excessive alcohol intake, anaemia, impaired renal and liver function,^40^ which could change during follow‐up period. Therefore, the bleeding risk of patients should be dynamically assessed for adjustment of therapeutic strategy and reduce of bleeding incidence.

This study has some limitations. First, due to the limited number of patients included, the incidence of major bleeding events was low and could not be analysed for pharmacogenomics. And the genetic SNPs of major bleeding events need to be validated in a larger cohort of rivaroxaban‐treated patients. Second, this study was an exploratory analysis on SNPs suggestively associated with bleeding risk and peak anti‐FXa level of rivaroxaban. The effects of SNPs need to be validated in independent large cohorts in future studies. Third, this study analysed the influence of genetic polymorphisms on anti‐FXa level and bleeding risk of rivaroxaban. SNPs associated with rivaroxaban anti‐FXa level and bleeding risk should be further studied in other FXa inhibitors including apixaban and edoxaban.

## 5 CONCLUSIONS

We first analysed the genetic factors related to peak anti‐FXa level and risk of bleeding events in patients with NVAF receiving rivaroxaban. *SUSD3* rs76292544 was suggestively associated with 12‐month bleeding events and five SNPs (*NCMAP* rs4553122, *PRF1* rs885821, *PRKAG2* rs12703159, rs13224758 and *POU2F3* rs2298579) were suggestively associated with peak anti‐FXa level.

## AUTHOR CONTRIBUTIONS

Conception and design: Yimin Cui and Qian Xiang, Jie Jiang. Provision of study materials or patients: Qian Xiang, Zhe Wang, Zhiyan Liu, Qiufen Xie, Guangyan Mu, Shuang Zhou, Hanxu Zhang, Zining Wang, Jie Jiang, Kun Hu, Yatong Zhang, Zinan Zhao, Dongdong Yuan, LiPing Guo, Tingting Wu, Jinhua Zhang, Na Wang, Jing Xiang, Zhichun Gu and Jianjun Sun. Collection of data: Qian Xiang, Zhe Wang, Guangyan Mu, Zhiyan Liu and Qiufen Xie. Data analysis and interpretation: Qian Xiang, Zhe Wang, Zhiyan Liu, Guangyan Mu. Manuscript writing: Qian Xiang and Zhe Wang. All the authors have read and approved the final manuscript.

## FUNDING

This work was supported by grants from the National Key R&D Program of China (2016YFC0904900), National Natural Science Foundation of China (81872940, 81973395 and 82073935) and Beijing Municipal Commission of Science and Technology of China Pharmaceutical Innovation Cultivation and Industry Support Platform Capacity Construction Project (Z191100007619038).

## CONFLICT OF INTEREST STATEMENT

The authors declare no relevant conflicts of interest.

## ETHICS STATEMENT

The study protocol was approved by the independent ethics committee of Peking University First Hospital and all subcentral hospitals. This study follows the principles of the Declaration of Helsinki. All patients who participated in this study provided informed consent.

## Supporting information

Supporting Information

## Data Availability

The data that support the findings of this study are deposited in the National Population Health Data Center (https://www.ncmi.cn) and available from the corresponding author upon reasonable request (DOI:10.12213/11.A0028.202009.338.V1.0).
