# Supplementary material for: Genetic variants influenced the risk of bleeding and pharmacodynamics of rivaroxaban in patients with nonvalvular atrial fibrillation: A multicentre prospective cohort study
Source: Clin Transl Med. 2023 May 18;13(5):e1263. doi: 10.1002/ctm2.1263 (PMC10196221; doi:10.1002/ctm2.1263)

**Supplementary Appendix**

[1. Participant eligibility criteria 1](#_Toc17554)

[2. Appendix Table 1 Univariate analysis for the association of baseline characteristic and 12-month cumulative bleeding events 2](#_Toc18251)

[3. Appendix Table 2 Cox analysis for baseline characteristic and 12-month cumulative bleeding events 3](#_Toc31926)

[4. Appendix Table 3 Univariate and Multivariate analysis for the association of baseline characteristic and peak anti-FXa activity. 4](#_Toc929)

[5. Appendix Table 4 Candidate genes of rivaroxaban 5](#_Toc6544)

[6. Appendix Figure 1 Flow diagram of enrolled participants 6](#_Toc14112)

[7. Appendix Table 5 Association of candidate genetic variation with peak anti-FXa activity and 12-month cumulative bleeding events 7](#_Toc25098)

[8. Appendix Table 6 Effects of suggestive genetic variations on both peak anti-FXa activity and 12-month cumulative bleeding events. 8](#_Toc12652)

[9. Appendix Figure 2 Functional implication of SNPs related with rivaroxaban based on GTEx database. 11](#_Toc528)

1. **Participant eligibility criteria**

The diagnostic criteria for nonvalvular AF (NVAF):

The diagnosis of atrial fibrillation requires rhythm documentation using 12-lead electrocardiogram, rhythm strip, pacemaker/ICD electrogram, or Holter electrocardiogram showing absolutely irregular RR intervals and no discernible distinct P waves. The duration of atrial fibrillation should be at least 30 seconds.

1. **Appendix Table 1 Univariate analysis for the association of baseline characteristic and 12-month cumulative bleeding events**

|  | **Group** | **OR (95%CI)** | ***P* value** |
| --- | --- | --- | --- |
| Age | ≥65 vs. <65 | 0.87 (0.44-1.73) | 0.262 |
| Male sex (%) | Female vs. Male | 1.04 (0.56-1.94) | 0.900 |
| BMI | <20 vs. ≥20 | 0.70 (0.18-2.73) | 0.606 |
| Dose-mg | <20 vs. ≥20 | 1.11 (0.42-2.95) | 0.612 |
| MPV-fl | ≥10 vs. <10 | 0.37 (0.19-0.71) | 0.003 |
| HGB-g/L | ≥110 vs. <110 | 0.62 (0.16-2.38) | 0.484 |
| Platelet count-10^9^/L | ≥100vs. <100 | 0.63 (0.06-6.15) | 0.688 |
| ALT-IU/L | ≥40 vs. <40 | 0.96 (0.28-3.31) | 0.943 |
| AST-IU/L | ≥40 vs. <40 | 1.10 (0.31-3.93) | 0.881 |
| Creatinine clearance-mL/min | ≥80 vs. <80 | 1.19 (0.64-2.21) | 0.589 |
| Hypertension (%) | Yes vs. No | 0.49 (0.26-0.94) | 0.033 |
| Diabetes mellitus (%) | Yes vs. No | 0.79 (0.40-1.58) | 0.505 |
| Hyperlipidemia (%) | Yes vs. No | 0.93 (0.48-1.77) | 0.814 |
| Heart failure (%) | Yes vs. No | 0.40 (0.14-1.13) | 0.084 |
| Prior stroke (%) | Yes vs. No | 1.08 (0.52-2.28) | 0.831 |
| ACEI/ARB (%) | Yes vs. No | 0.73 (0.39-1.38) | 0.338 |
| Calcium channel blocker (%) | Yes vs. No | 0.48 (0.24-0.96) | 0.037 |
| Beta-blocker (%) | Yes vs. No | 0.95 (0.50-1.78) | 0.864 |
| Nitrates (%) | Yes vs. No | 0.18 (0.02-1.41) | 0.102 |
| Antiplatelet therapy (%) | Yes vs. No | 1.06 (0.52-2.19) | 0.869 |
| Bleeding history (%) | Yes vs. No | 1.97 (0.47-8.15) | 0.347 |
| HAS-BLED | ≥3 vs. <3 | 1.82 (0.92-3.61) | 0.087 |

OR: Odds ratio; CI: Confidence interval; BMI: Body Mass Index; Dose: Dose of rivaroxaban; HGB: Haemoglobin; MPV: Mean platelet volume; ALT: Alanine aminotransferase; AST: Aspartate aminotransferase; ACEI: angiotensin-converting enzyme inhibitor; ARB: Angiotensin receptor blockers

1. **Appendix Table 2 Cox analysis for baseline characteristic and 12-month cumulative bleeding events**

|  | **Group** | **HR (95%CI)** | ***P* value** |
| --- | --- | --- | --- |
| MPV-fl | ≥10 vs. <10 | 0.54 (0.31-0.95) | 0.031 |
| Hypertension (%) | Yes vs. No | 0.59 (0.32-1.08) | 0.086 |
| Heart failure (%) | Yes vs. No | 0.54 (0.22-1.36) | 0.194 |
| Calcium channel blocker (%) | Yes vs. No | 0.62 (0.32-1.21) | 0.163 |
| Nitrates (%) | Yes vs. No | 0.22 (0.03-1.56) | 0.128 |
| HAS-BLED | ≥3 vs. <3 | 2.25 (1.31-3.89) | 0.004 |

HR: Hazard ratio; CI: Confidence interval

1. **Appendix Table 3 Univariate and Multivariate analysis for the association of baseline characteristic and peak anti-FXa level.**

|  | **Group** | **N** | **Mean±SD** | **Univariate analysis*-P* value** | **Multivariate analysis*-P* value** |
| --- | --- | --- | --- | --- | --- |
| Dose-mg | 5  10  15  20 | 1  30  89  131 | 227.70±0.00  223.04±106.08  298.72±125.01  267.98±137.00 | 0.042 | 0.035 |
| Age-yr | <65  ≥65 | 84  167 | 245.51±130.21  287.33±129.31 | 0.017 | 0.014 |
| Sex | Male  Female | 131  120 | 255.85±12.61  292.43±134.28 | 0.027 | 0.039 |
| BMI | ≥20  <20 | 235  16 | 271.27±130.69  303.66±133.76 | 0.339 | 0.438 |
| MPV-fl | ≥10  <10 | 128  123 | 239.10±122.59  308.97±118.32 | <0.001 | <0.001 |
| Creatinine clearance-ml/min | ≥80  <80 | 137  114 | 267.82±132.17  279.96±129.52 | 0.465 | 0.472 |
| Prior stroke | No  Yes | 205  46 | 270.86±134.50  284.38±113.88 | 0.528 | 0.837 |
| Antiplatelet therapy | No  Yes | 202  49 | 268.03±134.45  295.22±113.48 | 0.192 | 0.304 |

Dose: Dose of rivaroxaban; BMI: Body Mass Index; MPV: Mean platelet volume

1. **Appendix Table 4 Candidate genes of rivaroxaban**

| **Candidate genes** | **SNPs** | **Association results** |
| --- | --- | --- |
| *ABCB1* | rs4148738 | Concentrations of rivaroxaban |
|  | rs2032582 | Exposure to rivaroxaban;  Not associated with thromboembolism |
|  | rs1045642 | Exposure to rivaroxaban; Clearance of rivaroxaban;  Risk of thromboembolism |
|  | rs4728709 | Clearance of rivaroxaban |
|  | rs3842 | Risk of hemorrhage |
| *APOB* | rs13306198 | Risk of hemorrhage |
| *ABCA6* | rs7212506 | Risk of hemorrhage |

1. **Appendix Figure 1 Flow diagram of enrolled participants**

Basic information collection, including sex, age, BMI, previous medical history, combined medication, etc

Whole-exome sequencing

Peak anti-FXa level test

Blood was not extracted for peak anti-FXa activty and whole-exome sequencing (*N*=13).

Enrolled in study (*N*=257)

Patients with atrial fibrillation receiving rivaroxaban (*N*=270)

Obtaining informed consent (*N*=270)

1. **Appendix Table 5 Association of candidate genetic variation with peak anti-FXa activity and 12-month cumulative bleeding events**

| **SNPs** | **Gene** | **Participant** | **GENO** | **Peak anti-Xa**  **/ng·mL-1** | **Peak anti-Xa**  **-P value** | **Incidence of 12-month bleeding event** | **Incidence of 12-month bleeding event**  **-P value** |
| --- | --- | --- | --- | --- | --- | --- | --- |
| rs1045642 | *ABCB1* | 37 | AA | 288.66 | 0.748 | 26.9% | 0.092 |
|  |  | 127 | AG | 271.45 |  | 34.8% |  |
|  |  | 86 | GG | 270.09 |  | 37.3% |  |
| rs2032582 | *ABCB1* | 39 | AA | 294.11 | 0.906 | 29.6% | 0.527 |
|  |  | 89 | AC | 272.19 |  | 27.0% |  |
|  |  | 48 | CC | 263.44 |  | 42.4% |  |
|  |  | 38 | AT | 263.04 |  | 44.8% |  |
|  |  | 30 | CT | 280.28 |  | 35.0% |  |
|  |  | 6 | TT | 272.97 |  | 40.0% |  |
| rs13306198 | *APOB* | 223 | GG | 272.36 | 0.686 | 32.7% | 0.250 |
|  |  | 27 | GA | 283.18 |  | 50.0% |  |
| rs7212506 | *ABCA6* | 54 | CT | 269.76 | 0.812 | 40.6% | 0.056 |
|  |  | 196 | TT | 274.56 |  | 32.4% |  |

1. **Appendix Table 6 Effects of suggestive genetic variations on both peak anti-FXa level and 12-month cumulative bleeding events.**

| **SNP** | **Allele** | **Gene** | **Participant**  **(WH/HTZ/MT)** | **Peak anti-Xa /ng·mL^-1^**  **(WH/HTZ/MT)** | ***P* value-**  **peak anti-FXa** | **Incidence of 12-month bleeding event (WH/HTZ/MT)** | ***P* value-**  **12-month bleeding events** |
| --- | --- | --- | --- | --- | --- | --- | --- |
| rs640198 | T>G | *MMP13* | 60/117/73 | 258.9/263.1/302.3 | 0.020 | 13.9%/33.7%/49.1% | 0.001 |
| rs14221 | A>C | *GOT2* | 33/109/108 | 313.9/276.9/257.7 | 0.005 | 41.7%/40.7%/25.0% | 0.033 |
| rs733447 | G>A | *RIN3* | 135/96/19 | 263.9/272.8/345.7 | 0.017 | 25.3%/41.3%/63.6% | 0.006 |
| rs2304386 | C>G | *RYR3* | 221/29/0 | 282.4/205.8/*NA* | 0.006 | 36.7%/15.8%*NA* | 0.043 |
| rs38409 | T>C | *GGCT* | 42/99/109 | 221.3/276.9/290.6 | 0.006 | 19.4%/31.5%/43.8% | 0.012 |
| rs8106303 | A>C | *FXYD5* | 13/71/166 | 210.2/262.4/283.2 | 0.009 | 11.1%/25.0%/40.0% | 0.020 |
| rs629990 | G>A | *TNNT3* | 5/35/210 | 398.7/292.0/267.5 | 0.011 | 80.0%/47.4%/31.4% | 0.018 |
| rs876840 | G>C | *MLF1IP* | 24/105/121 | 245.5/263.4/287.9 | 0.011 | 22.2%/27.4%/43.0% | 0.043 |
| rs11146363 | C>T | *PWWP2B* | 42/111/97 | 255.6/265.6/290.4 | 0.011 | 32.3%/23.0%/47.2% | 0.045 |
| rs28540767 | C>T | *HERC2P2* | 197/53/0 | 262.2/315.8/*NA* | 0.012 | 29.7%/51.3%/*NA* | 0.019 |
| rs708165 | T>C | *C12orf71* | 224/25/1 | 267.0/319.8/579.8 | 0.013 | 31.6%/55.6%/100% | 0.046 |
| rs7252937 | A>G | *DOCK6* | 207/43/0 | 281.9/233.1/*NA* | 0.013 | 38.5%/13.8%/*NA* | 0.023 |
| rs2736982 | G>A | *DSPP* | 20/96/134 | 312.4/284.3/260.0 | 0.014 | 52.9%/39.7%/27.2% | 0.013 |
| rs38414 | G>A | *GGCT* | 45/100/105 | 226.6/275.4/291.9 | 0.016 | 20.6%/31.0%/44.4% | 0.014 |
| rs34224564 | G>C | *STMND1* | 140/95/15 | 252.8/302.6/283.1 | 0.017 | 27.3%/41.4%/62.5% | 0.025 |
| rs4743163 | C>A | *TRIM14* | 90/117/43 | 274.1/270.8/238.0 | 0.018 | 44.0%/29.5%/20.8% | 0.028 |
| rs12501626 | C>A | *ENPEP* | 141/96/13 | 261.5/282.3/339.4 | 0.019 | 29.2%/37.5%/66.7% | 0.040 |
| rs8110220 | T>C | *HSD17B14* | 182/65/3 | 281.9/255.8/151.0 | 0.020 | 38.6%/23.8%/0.0% | 0.048 |
| rs4361282 | C>G | *FRMD4B* | 139/97/14 | 258.3/292.3/295.3 | 0.022 | 27.4%/43.7%/36.4% | 0.041 |
| rs10818475 | C>G | *TRIM14* | 105/110/35 | 294.2/263.3/243.4 | 0.027 | 41.4%/31.4%/15.0% | 0.024 |
| rs11967042 | G>A | *LAMA2* | 98/121/31 | 291.9/268.9/233.7 | 0.030 | 43.1%/30.1%/22.7% | 0.024 |
| rs1126758 | C>T | *PAH* | 215/34/0 | 278.7/247.3/*NA* | 0.031 | 38.0%/14.8%/*NA* | 0.038 |
| rs3734491 | G>A | *TUBB2A* | 213/35/1 | 269.4/303.7/135.1 | 0.031 | 31.6%/54.5%/*NA* | 0.022 |
| rs7252466 | T>C | *ACSBG2* | 32/112/106 | 239.7/265.5/292.2 | 0.031 | 28.6%/26.8%/44.6% | 0.036 |
| rs30842 | A>C | *GOT2* | 26/103/121 | 312.3/276.1/263.0 | 0.032 | 31.3%/45.0%/24.7% | 0.043 |
| rs3199064 | T>G | *NANS* | 110/110/30 | 294.0/262.7/237.9 | 0.034 | 42.7%/27.8%/18.8% | 0.016 |
| rs11127132 | C>T | *BRE* | 31/113/106 | 346.2/262.1/264.4 | 0.035 | 30.0%/36.4%/26.3% | 0.014 |
| rs1130529 | C>T | *PLOD1* | 118/108/24 | 253.9/291.1/291.3 | 0.036 | 32.5%/31.2%/58.8% | 0.049 |
| rs12261006 | C>T | *ACBD7* | 220/29/1 | 267.7/313.5/384.6 | 0.039 | 31.8%/52.6%/100% | 0.023 |
| rs2255317 | C>T | *MATN2* | 154/85/11 | 265.0/284.8/305.2 | 0.041 | 27.1%/41.7%/70.0% | 0.005 |
| rs2073368 | G>T | *ITSN1* | 122/105/23 | 253.8/295.5/277.7 | 0.042 | 23.2%/40.3%/61.1% | 0.006 |
| rs59348778 | G>C | *SGSM2* | 219/31/0 | 279.1/234.3/*NA* | 0.042 | 37.3%/10.5%/*NA* | 0.049 |
| rs679620 | C>T | *MMP3* | 36/110/104 | 326.2/264.8/264.5 | 0.043 | 50.0%/38.2%/25.3% | 0.026 |
| rs7752421 | A>T | *SNAP91* | 66/141/43 | 288.9/276.3/240.7 | 0.044 | 35.7%/40.0%/13.3% | 0.045 |
| rs3108200 | A>G | *ZNF567* | 132/93/25 | 256.7/288.4/306.9 | 0.045 | 26.5%/44.3%/44.4% | 0.041 |
| rs3736456 | T>C | *CYP4V2* | 122/109/29 | 255.3/284.6/302.4 | 0.047 | 26.0%/38.8%/50.0% | 0.029 |
| rs3831339 | .>T | *ESD* | 106/112/32 | 266.1/264.6/329.2 | 0.047 | 20.3%/45.3%/36.4% | 0.037 |
| rs1049402 | G>C | *GARS* | 42/105/103 | 235.6/273.5/289.0 | 0.048 | 26.7%/27.6%/45.1% | 0.032 |
| rs6728818 | G>A | *COL6A3* | 57/112/72 | 295.0/272.1/258.7 | 0.048 | 40.0%/42.7%/18.2% | 0.028 |

MH: mutated homozygous; HTZ: heterozygous; WH: wild homozygous

**GGCT* rs38410, rs40237, and rs38414 had strong linkage disequilibrium. *TNNT3* rs629990, rs2292473, and rs2292471 had strong linkage disequilibrium. *MLF1IP* rs902174 and rs876840 had strong linkage disequilibrium. *DOCK6* rs12609039 and rs7252937 had strong linkage disequilibrium. *C12orf71* rs708167 and rs708165 had strong linkage disequilibrium. *MATN2* rs2255317 and rs35312215 had strong linkage disequilibrium. *SGSM2* rs60072521 and rs59348778 had strong linkage disequilibrium. *MMP3* rs679620, rs591058, and rs602128 had strong linkage disequilibrium. *ACBD7* rs185878561 and rs12261006 had strong linkage disequilibrium. *DSPP* rs2736982 and rs13131929 had strong linkage disequilibrium. *LAMA2* rs11967042 and rs372962551 had strong linkage disequilibrium.

1. **Appendix Figure 2 Functional implication of SNPs related with rivaroxaban based on GTEx database.**


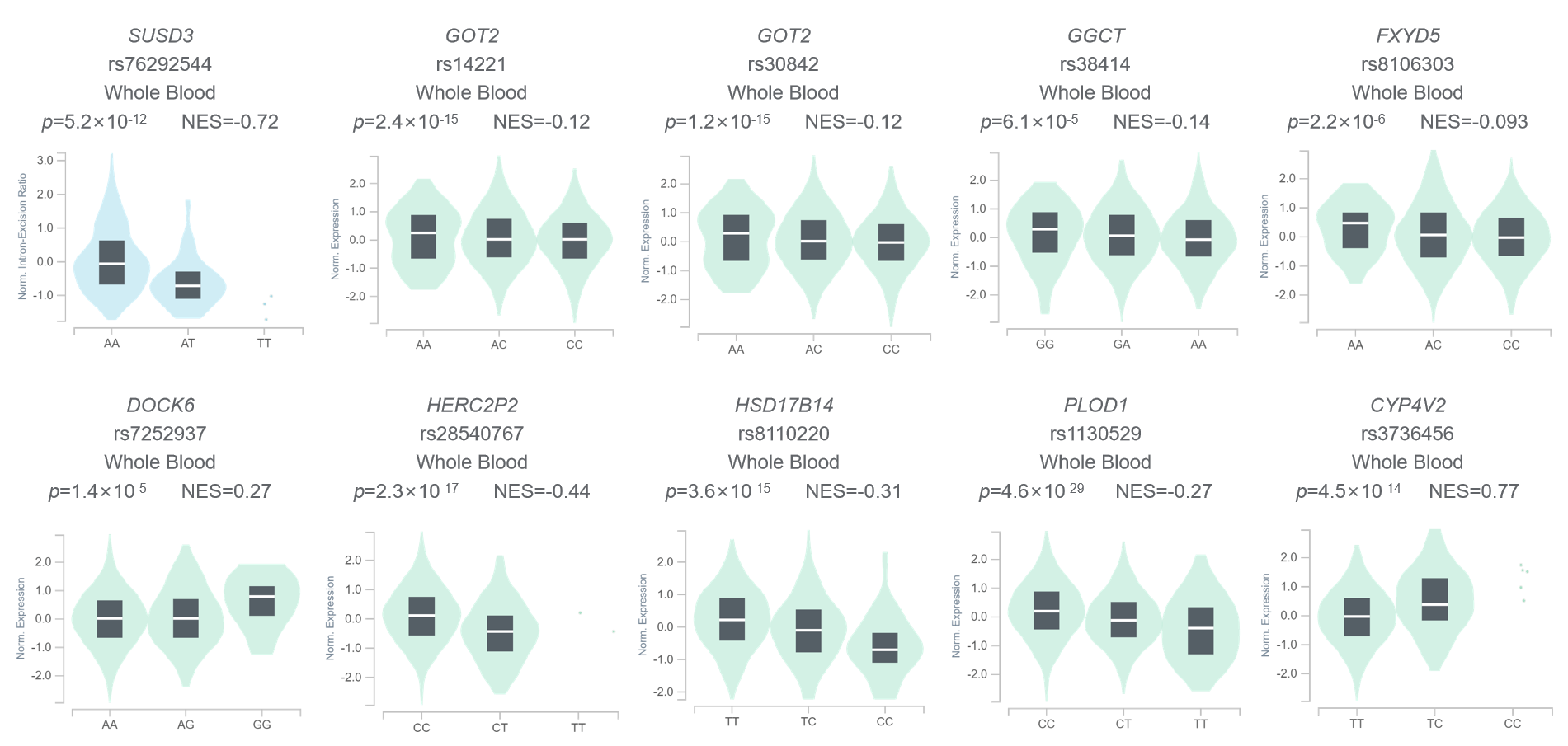

Supplement: Supplementary file 1 — Supporting Information [file CTM2-13-e1263-s001.docx]
